# Correction: SH2 Modified STAT1 Induces HLA-I Expression and Improves IFN-γ Signaling in IFN-α Resistant HCV Replicon Cells

**DOI:** 10.1371/annotation/ff8fe1fe-36ae-43fe-9350-8ba2f191331a

**Published:** 2010-11-17

**Authors:** Bret Poat, Sidhartha Hazari, Partha K. Chandra, Feyza Gunduz, Luis A. Balart, Xavier Alvarez, Yong Zhang, Michael J. Holtzman, Srikanta Dash

The authors wish to add two authors to the byline, Dr. Yong Zhang and Dr. Michael J. Holtzman. The correct byline is now: Bret Poat, Sidhartha Hazari1, Partha K. Chandra1, Feyza Gunduz, Luis A. Balart, Xavier Alvarez, Yong Zhang, Michael J. Holtzman, Srikanta Dash.

The affiliation for Drs. Zhang and Holtzman is: Department of Medicine, Department of Cell Biology Washington University School of Medicine, St. Louis, Missouri, United States of America.

The author contributions should now be: Conceived and designed the experiments: BP SH SD. Performed the experiments: BP SH PKC FG XA. Analyzed the data: BP SH PKC FG LAB SD. Contributed reagents/materials/analysis tools: BP SH PKC XA YZ MJH SD. Wrote the paper: BP SD. Critically read the manuscript: LAB.

